# Supplementary material for: Joint angle trajectories are Robust to segment length estimation methods in human reaching
Source: PLoS One. 2025 Nov 21;20(11):e0310475. doi: 10.1371/journal.pone.0310475 (PMC12637938; doi:10.1371/journal.pone.0310475)
Supplement: Attachment — Submitted filename: Response to Reviewers.pdf [file pone.0310475.s004.pdf]

Response to Reviewers

Reviewer #2: Thank you for addressing the comments, the paper is mainly clear. There is unfortunately still on critical point that is not entirely clear to me.
A. We thank the reviewer for the thorough review of our revision. We have addressed all points to clarify them to the best of our abilities as described below in detail and accepted all suggestions.

Major comments:
Q. Line 211-213: I do not understand how the between-participant standard deviation was calculated. Was it calculated separately for each participant or for all participants? If it is only one participant, how can it be a between-participant variability? It also does not make sense with the explanation in line 99, because there is no variation in body size in a single participant.

A. To clarify the calculation of within-subject and between-subject variability described in the Statistics and Reproducibility subsection, we added formulas on lines 212-229. We have clarified that the angles calculated using average segments are a proxy for a hypothetical participant of the same height, but with different segment lengths. We also mention that this is a conservative estimate of the between-subject variability due to the height-based variability being excluded.

Q. Furthermore, I do not agree with the decision to include the results of both methods in the calculation when the goal is to compare them. It might also help to add the number of trajectories that were used to help readers understand how the calculation was made.
A. There are multiple ways the comparisons can be made. The within-subject trial-to-trial variability in trajectories are related to not only limb geometry but also neural noise, i.e., the variability in the output of the nervous system motor control strategies. This is a confounding factor. The between-subject variability calculated as described in Methods does not include neural noise, because it compares each trial to its equivalent produced by a limb with different segment lengths. This metric only includes the variability in angles that is due to the changes in segment lengths. The number of trajectories was 15 as mentioned on lines 158-159.

Q. Figure 2: why is the presentation of SDw and SDb so different? Since they are very similar variables which were calculated in a similar way and the legend describes both similarly too, it seems that it would make sense to either use lines for both or histogram plots for both. Furthermore, the meaning of the + is not explained in the legend. I would

also recommend to use different colors than in Figure 2, because something different is plotted.
A. We have updated the figure to represent both metrics similarly, now in red and blue. The + are no longer plotted. We followed the recommendation to use different colors in Fig. 2 and 3. The red was kept the same as these are the same variables that are plotted.

Minor comments:
Q. I recommend using within-participant and between-participant for the variability.
A. Done, thanks!

Q. Line 96-98: this sentence is not grammatically correct
A. We have reworked this sentence to say: “We define redundancy in arm reaching as variability in the angular trajectories of joint degrees of freedom (DOFs).”

Q. Line 115: the overall height has increased, remove improvements in once
A. Done, thanks!

Q. Line 189: “to” should be added before “drive”?
A. Done, thanks!

Q. Line 197: directions should be direction
A. Done, thanks!

Q. Line 199: word missing (an)
A. Done, thanks!

Q. Line 213: "that" should be "the"?
A. Done, thanks!

Q. Line 247: limbs
A. Done, thanks!

Q. Line 263: remove comma after "that"

A. Done, thanks!
