# Supplementary material for: Joint angle trajectories are Robust to segment length estimation methods in human reaching
Source: PLoS One. 2025 Nov 21;20(11):e0310475. doi: 10.1371/journal.pone.0310475 (PMC12637938; doi:10.1371/journal.pone.0310475)
Supplement: Attachment — Submitted filename: Reviewer responce R5.pdf [file pone.0310475.s006.pdf]

Reviewer #3: Thank you for your detailed responses and revisions. The manuscript has been notably improved. To further enhance its methodological clarity, Please consider the following points, or provide a valid justification if not addressed:

1 The authors' rationale for focusing on global effects is noted. However, the key finding that segment scaling alters amplitude but not the shape of the trajectory is inherently a time-series claim . Relying on scalar summaries and visual inspection (figure 2) remains a sub-optimal level of evidence for this conclusion. The application of a 1D-SPM analysis would provide a direct and robust statistical validation that the trajectories are equivalent across their entire duration, thereby strengthening this central result.
A. We have performed the 1D-SPM, added a new figure summarizing the result and included descriptions of it in Methods and Results. No significant differences were found.

2) The phrasing in the Abstract (Lines 48-49) still attributes variability specifically to "how the nervous system selects," which is a strong causal claim not fully supported by kinematic data. We recommend rephrasing to more directly reflect the observed evidence, for example: "...indicating that the variability inherent in movement execution outweighs that introduced by morphological differences."
A. Done, thank you for spotting this.

3) To bolster methodological confidence, please cite existing literature where Simscape Multibody has been specifically validated for calculating human joint angles.
A. We have moved the reference to the publication validating this method of joint angle calculation to the beginning of the Data Analysis section.
