# Supplementary material for: Joint angle trajectories are Robust to segment length estimation methods in human reaching
Source: PLoS One. 2025 Nov 21;20(11):e0310475. doi: 10.1371/journal.pone.0310475 (PMC12637938; doi:10.1371/journal.pone.0310475)
Supplement: Attachment — Submitted filename: PlosOne_ResponseToReviewers.docx [file pone.0310475.s002.docx]

**Response to reviewers**

Our responses to the editor and reviewers are presented in **bold writing**. All page numbers cited in our responses refer to the ‘clean’, revised manuscript.

**Reviewer #1**

Q. I noticed in many cases bulk citations that should be avoided, e.g. at line 51 you cite [2-9]. At line 164 you have {Citation}.

**A. Thank you for bringing this to our attention. We have revised the corresponding portions of the Introduction and Discussion to elaborate on relevant findings from previous studies and corrected the error.**

Q. The paper does not present the statistical analysis that was made. It is mentioned ANOVA method, but without details.

**A. The description of ANOVA was in the last paragraph of the Methods section immediately following the description of RMSE, now on lines 163-177. We have added a section title, Statistics and Reproducibility, to highlight it.**

**Reviewer #2**

Q. This paper describes an experiment where the goal was to calculate joint angles with a musculoskeletal model that was personalized using an Average method and an Individual method in an experiment that used virtual reality. While I believe that the study could be interesting, at this point the manuscript misses a lot of vital information that is required to understand what was being done. Therefore, I cannot recommend publication of the study at this point.

**A. Thank you for the supportive feedback. We have detailed the changes to the concerns and comments below.**

Q. The text is so compact in the manuscript that it is a bit difficult to read the paper. It would be great if the text is double spaced.

**A. Thank you for the suggestion. We have changed the line spacing of the main text for your additional review and updated all formatting to match the PLOS One guidelines.**

Q. It would also be great if the raw experimental data could be shared as well.

**A. We have added the raw experimental data to the Figshare repository. The data includes the joint angle timeseries calculated with the two methods per target per subject per degree of freedom.**

Q. Abstract: 8 out of 20 lines are a rather general introduction, which does not explain why the authors were specifically interested in testing different scaling methods. Furthermore, the methods are not described clearly: which motion capture technology is being tested? What sort of tasks were performed? How many participants? Also, it would be great if some quantitative results were added.

**A. Thank you for the suggestion. We have added the requested information into the abstract and rewrote it to include more details.**

Q. Introduction: while I generally liked the introduction, a statement/final paragraph is missing which describes exactly the contribution of the study that is described in the manuscript. Therefore, it is really difficult to assess the usefulness of the methodology and the results towards the contribution. For example, why did the authors choose the specific tests that they describe in line 146-154.

**A. We have included additional clarifications and explicit statements about the goal of the study on lines 90 - 101.**

Q. Line 80: The authors could further expand on the redundancy as it is quite relevant to their study.

**A. We have followed this suggestion and expanded on redundancy now on lines 60 – 79.**

Q. Methods, line 114: please describe the type of virtual reality that was being used. Why is virtual reality relevant?

**A. Thank you for the suggestion. We have added more details on lines 110 – 124.**

Q. Methods: Please describe (here or in the introduction) how comparable the experimental setup is to the one that is expected to be used in the clinic

**A. Thank you for this point. We have expanded on how we envision this technology can be used for future clinical assessment in Discussion on lines 240-252.**

Q. Methods: please describe the experiment and data processing more clearly: what tasks were measured? How many repetitions? What was done to the measured data exactly?

**A. We have expanded the description in Methods on lines 110-137.**

Q. Methods: I generally did not understand the data analysis section. The authors have described it a bit too densely. For example, it is only described that the “Average method” relies “on published anthropomorphic dimensions as fractions of individual height”, but how were these applied? And which segment lengths were changed in the “Individual method”. Why was a time-independent standard deviation used?

**A. We have expanded the Data Analysis section in Methods now on lines 140-160.**

Q. Results: there are more results mentioned in table 1 than just the Average and Individual method. Please explain where these values come from. For table 2, since the tasks are not described, it is difficult to understand what the ROM means.

**A. We have expanded the description of Table 1 in Results on lines 179-192 and of Table 2 on lines 193-208. We have also added the active ROM calculated from the reaching movements in addition to the maximal ROM and added RMSE ranges for each DOF in Table 2.**

Q. Discussion, line 197-198: I do not understand this sentence. According to the methods, there were two methods and there is not a separate ground truth, so how can one “alter the values the most”.

**A. This sentence was removed, instead, we expanded on the clinical applicability of our findings in the Discussion on lines 221-252.**

Q. Discussion, line 211-212: what is “good enough” based on?

**A. This statement was removed.**

Q. A concluding paragraph is missing.

**A. We added a concluding paragraph on lines 253-264.**
