# Supplementary material for: Joint angle trajectories are Robust to segment length estimation methods in human reaching
Source: PLoS One. 2025 Nov 21;20(11):e0310475. doi: 10.1371/journal.pone.0310475 (PMC12637938; doi:10.1371/journal.pone.0310475)
Supplement: Attachment — Submitted filename: Review responces.docx [file pone.0310475.s003.docx]

We thank the reviewer for their due diligence. The pointed questions have made us rethink how we calculate the variability, and the resulting changes in methodology described below have further improved our results (Fig. 3). Our answers are marked with A and the new text copied from the manuscript is marked with red.

Reviewer #2:

*Q. I am not sure if the data that is made available meets the standard required by PLOS One, as it is only the root-mean-square errors and no raw data, or perhaps time series data of joint angles over time. E.g., Figure 2 cannot be reproduced as far as I can see.*

A. We thank the reviewer for spotting this inadequacy of data sharing. We have rectified it by sharing the joint angles normalized in time between onset and offset of movement calculated with the two methods on Figshare <https://doi.org/10.6084/m9.figshare.28633340.v1>

Major Comments

*Q. Line 28-30: This sentence does not describe the purpose of the paper correctly. As far as I understood, the goal of the paper is to investigate if joint angles can be estimated from a model scaled from average body proportions as accurately as when using a model with measured body proportions.*

A. Thank you for identifying this, we have reworked the abstract to have sections and modified the Background section as follows: “Quantitative movement analysis is increasingly used to assess motor deficits, but joint angle calculations depend on assumptions about limb segment lengths. These lengths are often estimated from average anthropometric proportions rather than measured directly. The extent to which such assumptions influence joint angle accuracy and variability remains unclear.”

*Q. Line 56: the statement “obstacle to widespread adoption” is contradictory to line 51 which claims that there already is widespread adoption.*

A. We have changed the sentence now on lines 74-75 to say “gaining interest” to avoid contradiction.

*Q. Throughout (line 94/98/…): it is confusing to me that the word “variability” is used with at least two different meanings, the variability in segment length and the variability in reaching movement execution. Furthermore, it also seems to me that sometimes “variability in segment lengths” is used to describe the fact that there is variation in measurements, not necessarily a difference in the actual segment length (e.g., in line 166 and 176). In that case “model/measurement inaccuracy” would be more fitting. But also between variability in segment length and movement execution, I think that the readability could be improved if different words are used to describe these different meanings, although I am not sure what that other word should be.*

A. We agree that this is confusing. We have also realized that the way we calculated the two metrics, as standard deviation and RMSE, may have exaggerated the differences between them. We now calculate differences between due to the two methods of segment length estimation between the same individual reaches (not averages as before) and quantify variability as standard deviation as described in detail in Methods. Briefly on lines 208-213: “Statistical analysis compared the within- and between-subject variability. The within-subject variability for a given reaching movement was calculated as the standard deviation (SDw) of the angular trajectory of each DOF from simulations with individually measured segments (Fig. 2, red shaded areas). The between-subject variability was calculated as the standard deviation (SDb) between the corresponding angular trajectories from simulations with averaged and individual segments.” This labelling of within- and between-subject variability as SDw and SDb hopefully makes things more clear. We also indeed see lower values of SDb (Fig. 3).

*Q. Line 166-169: I do not understand the explanation of the RMSE calculation. Since there is a “between” in the sentence, I expected to see and “and” to indicate the second item between which the RMSE is calculated. Is it calculated as the error between the two methods, meaning that one of the models is considered a ground truth? Or is there a separate ground truth that both are compared to?*

A. We have changed calculations to standard deviation as mentioned in answer to previous question. The detailed description is in the Statistics and Reproducibility section.

Q. Methods: a section/paragraph describing the analysis approach is missing. Therefore, the relevance of the variables mentioned in the results is not clear to me. Please add a section describing the analysis approach.

A. We have clarified the rationale for the analysis in Introduction on lines 89-95 and 122135. The Methods are updated in the Statistics and Reproducibility section.

Q. Lines 177-185: this piece of text should be in the discussion or the methods.

A. Done, we moved this into Methods right before Statistics and Reproducibility section.

Q. Line 191: is this based on literature or based on the current paper’s results? I also do not understand what is meant with “a small portion”. How is that defined?

A. We revised this sentence now on lines 240-241 as follows: “The ranges of motion observed in major arm DOFs during the recorded reaching movements comprised a fraction of the joints’ anatomical maximum range of motion (Table 2).”

Q. Line 202-203: please rephrase. An ANOVA test cannot show similarity, it can only show difference or lack of evidence for a difference. A correlation analysis should be used to test for similarity.

A. We revised this section because we changed how the two metrics are calculated. Now ANOVA shows significant differences, i.e., the between-subject standard deviation in trajectory differences calculated between the two types of segment length metrics is lower than the typica trial-to-trial variability of those trajectories. The detailed statistical results are summarized on lines 249-258.

Q. Line 214: while I agree with the statement, a conclusion should not be in the results section

A. We removed this sentence from Results.

Q. Line 240-241: Was this based on literature? The current study did not show this in my opinion. Please add a citation or rephrase.

A. We removed this speculative section from our Discussion. We now discuss redundancy on lines 277-282: “Our findings indicate that morphological variability between individuals contributes less to joint angle variability than the variability expressed by individuals across repeated movements. This suggests that within-subject variability arises primarily from how the nervous system selects among redundant motor solutions, rather than from differences in body morphology, consistent with the idea of optimal control of movement. However, neural noise, sensory uncertainty, or inconsistent control strategies may also contribute to the observed between-subject variability.”

Q. Line 251: how do the authors conclude a measurable effect when there was no statistically significant difference?

A. Well spotted! We have revised the concluding paragraph now in lines 283-292 to reflect our updated results better: “In summary, our findings show that assumptions about segment length have minimal impact on joint angle trajectories during reaching, with any small variations reflecting kinematic redundancy rather than morphological differences. These variations were far smaller than the natural within-subject variability observed across repeated movements for most indivduals, indicating that movement variability arises primarily from how the nervous system selects among redundant motor solutions. Importantly, segment length estimation methods did not alter the shape of joint angle trajectories, suggesting that clinical assessments of movement quality or range of motion are unlikely to be affected. Together, these results highlight that variability in motor performance is driven more by neural control processes than by body morphology, though factors such as neural noise and sensory uncertainty may also contribute.”

Q. Table 1: between which two variables is the difference (final column) defined?
A. The previous two columns. We have added a note under the table as follows: “the Difference was calculated between lengths measured with Individual and Average methods.”

Q. Table 2: shouldn’t there be results for both methods in this table? (related to the missing section in the methods about the analysis approach)

A. Done, data added.

Q. Table 2: How is the active ROM defined? And the maximal ROM? (could also be in an analysis section)

A. We have added the details of those calculations and the source in Methods on lines 196-199.

Minor Comments
Q. Line 33: MATLAB

A. Fixed.

Q. Line 48-50: it would be great if the authors could add what the Fugle-Meyer score investigates.

A. We stated that it measures post-stroke impairment.

Q. Line 50: I believe that measures should be measure (metrics measure)

A. Fixed.

Q. Line 71-72: I find it confusing that this line cites ref. [6] while the experiment in ref. [9] is described.

A. This is corrected, the paper that reported on the original data collection of the data used in this study is cited.

Q. Line 104: consider writing out West Viriginia for non-US-based readers

A. Done.

Q. Line 110 (and onwards) consider using participants instead of subjects: (<https://pubmed.ncbi.nlm.nih.gov/38075407/>)

A. Done. Although, we did use terms such as within-subject and between-subject variability as these are more established terms than within-participant and between-participant ones.

Q. Line 164: for readability, I would explicitly mention that the movement ended once they returned to the centre position.

Q. Done; now on line 214-15.

Q. Line 224: it was not clear until here that active and passive range of motion were important for the study. Consider including in the introduction or methods, earlier in the manuscript.

A. Done, we added a mention in Introduction on lines 69-71: “Movement analysis commonly relies on practitioner exploration of active or passive range of motion of specific joints. For more complex movements, subjective observation or rating scales are utilized.”

Q. Line 218-229: I was wondering if the study population of the paper included especially short or tall people, and if the authors considered their population reflective of the general population. This would be an interesting discussion topic/limitation.

A. We believe that our cohort was typical of our current average height of population. Last century, when most of the common ratios were derived, the average population was shorter. That is why we included the earlier study that measured segment lengths and individual heights to see if the results generalize to the current taller population. We have added these thoughts to Introduction on lines 114-121, Results on lines 232-234, and Discussion lines 261-263.
