# Supplementary material for: Joint angle trajectories are Robust to segment length estimation methods in human reaching
Source: PLoS One. 2025 Nov 21;20(11):e0310475. doi: 10.1371/journal.pone.0310475 (PMC12637938; doi:10.1371/journal.pone.0310475)
Supplement: Attachment — Submitted filename: Reviewer Comments R4.pdf [file pone.0310475.s005.pdf]

Additional Editor Comments:

The reviewer recommends substantial further revisions before acceptance. They request clearer justification of the study's practical relevance and methodological rigor, including identification of applications for the Average (body-height) method, a justification or power analysis for the small sample size, and rationale for the chosen motion capture system. Additional validation steps are required such as comparing Simscape Multibody results to standard biomechanical tools (e.g., OpenSim) and applying time-series statistics (1D-SPM) to the angular data. The reviewer also asks for better-qualified interpretations, particularly regarding neural versus morphological variability, and for clear limitations stating that the results apply only to healthy upper-limb movements, not to clinical populations or other tasks like gait.

A. All reviewer critiques were answered as detailed below.

Review Comments to the Author

Reviewer #3:

1. The manuscript should clearly mention specific applications where the Average (body-height) method is commonly used, to better justify the practical relevance of this comparison.

A. We thank the reviewer for this thoughtful suggestion. We have revised the Introduction and Discussion sections to explicitly highlight several common applications where the Average (body-height) method is routinely used. Specifically, we now mention that anthropometric scaling based on body height is widely applied in ergonomic design and workplace safety, Markerless motion capture systems (e.g., Kinect, OpenPose) and biomechanical modeling platforms such as OpenSim, and remote clinical assessments and tele-rehabilitation, see lines 76-78 and 81-86.

These examples reinforce the practical relevance of evaluating the accuracy and variability introduced by the Average method. By demonstrating that the resulting joint angle trajectories remain robust, even when using generalized anthropometric estimates, our findings support the use of this method in both research and clinical settings where individual segment measurements are not feasible. We have added these clarifications in the revised manuscript in Abstract, Introduction, Results, and Discussion.

2. The sample size of nine participants is too small to draw generalizable conclusions regarding anthropometric variability and joint angle calculation; a power analysis or justification for sample size is needed.

3. The manuscript should address the lack of an a priori power analysis.

A. We appreciate the reviewer's concern regarding the sample size and power analysis. We respectfully clarify that our primary goal was not to generalize population-wide anthropometric variability, but rather to assess the relative impact of segment length estimation methods (Average vs. Individual) on joint angle trajectory calculations within individuals performing repeated movements. As such, the study was designed around a within-subjects experimental framework, where each participant served as their own control across two modeling conditions (lines 33–34, Methods section).

We acknowledge that nine participants may be considered modest for between-subject comparisons. However, below we highlight several points that support the validity and robustness of our conclusions.

Purpose of Study – Methodological Evaluation: This study evaluates *the effect of model scaling method* on joint angle estimates, not anthropometric variability across the general population. The segment proportions used in the Average method are derived from much larger cohorts (e.g., Dempster 1955; Canda 2009, cited in Table 1), and we compared these published proportions with our small but representative sample to confirm similarity (lines 213–219). The key finding that differences in segment scaling have a smaller effect on joint angle calculations than natural within-subject variability is robust to sample size, as it hinges on direct within-subject comparisons.

Effect Size and Variance Structure: The joint angle deviations introduced by segment length estimation (σ_between) were consistently smaller than the variability of repeated trials within the same individual (σ_within), as shown in Figure 3 and Table 2. This large and consistent effect size (with σ_between often <10% of σ_within across joints) makes the findings reliable even with a small N. These differences were statistically significant in the repeated-measures ANOVA (Table 3, lines 281-287), further supporting the strength of the observed effect.

Simulation-Based Approach and Standardization: Because the analysis used a physically accurate dynamic model and high-resolution motion capture with tightly controlled virtual reality–based reaching tasks, the data were inherently low-noise and standardized (Methods). This design increases the sensitivity of within-subject comparisons and reduces the need for large samples.

Post hoc Justification of Power: To address this critique directly, we have added a post hoc power analysis to this computational study using the observed effect size (Cohen's d ≈ 1.5–2.5 for the difference between σ_within and σ_between across DOFs). With this effect size and within-subject design, power exceeds 0.9 for detecting differences at $\alpha = 0.05$, supporting the adequacy of the sample size for our main comparisons. This information has been added to the revised Statistics and Reproducibility section (lines 249–254) and Results (lines 287–292).

Scope of Claims: we have revised the Conclusions to clarify that the findings demonstrate the robustness of joint angle calculations to segment scaling assumptions in the context of upper-limb reaching tasks, and should not be overgeneralized to lower limbs or all forms of movement (lines 323-326).

4. A short justification for the specific motion capture system used is required.

A. We included the justification in Methods on lines 168-173.

5. The use of self-reported height introduces possible measurement error that may affect scaling accuracy; direct measurement of height should be considered, or the effect of self-report error quantified.

A. We appreciate the reviewer's attention to the potential limitations of using self-reported height. In our study, height was used exclusively for calculating segment lengths in the Average method of model scaling, which relies on published anthropometric ratios (e.g., humerus = 0.188 × height). The scaling of individual segment lengths from height results in linear adjustments to segment dimensions. A typical self-report error is on the order of 1–2 cm (about 1% of height). Given that segment lengths are proportional to height, such small deviations would lead to changes of only ~1% in segment dimensions. As shown in our analysis (Fig. 2, Table 2), the joint angle deviations due to even 10% segment length differences were small—typically <2°—and were still much smaller than the within-participant variability observed across repeated movements (σ_w) (Fig. 3).

Moreover, the use of self-reported height reflects common practice in remote or large-scale studies, where direct measurement may not be feasible (e.g., telemedicine, markerless motion capture). Our goal was to evaluate the robustness of model scaling in such real-world scenarios. By demonstrating that joint angle calculations remain accurate even when segment lengths are inferred from self-reported height, our findings support the practical reliability of motion capture assessments in both clinical and non-laboratory settings.

6. The authors should apply a time-series statistical method (1D-SPM) to compare entire angular trajectories rather than only summary differences, because this will detect localized temporal differences that pointwise ANOVA on scalar summaries can miss.

A. We thank the reviewer for this valuable suggestion regarding the use of time-series statistical analysis such as one-dimensional statistical parametric mapping (1D-SPM). We agree that 1D-SPM is a powerful tool for identifying temporally localized differences along continuous joint angle trajectories, and it is particularly useful when comparing group-level effects or task conditions with meaningful temporal structure. However, we respectfully note that the primary goal of our study was not to detect localized deviations across the time course of angular trajectories, but rather to evaluate the global effect of segment length scaling methods on joint angle calculations in the context of redundant upper-limb reaching. Moreover, the temporal profiles of the joint excursions are constrained by the recorded marker trajectories, which are applied to both analyses. Therefore, the temporal profiles of the resulting joint angular trajectories would be unaltered, as now clarified on lines 275-276. Our approach—comparing within- and between-participant variability using time-normalized trajectories and computing scalar variability metrics (σw and σb)—was designed to quantify the overall magnitude of variability attributable to scaling assumptions across the full movement duration.

7. The authors used Simscape Multibody instead of standard musculoskeletal modeling software (e.g., OpenSim) but did not provide validation or comparison to ensure that the computed joint angles are biomechanically consistent.

A. We appreciate the importance of cross-software validation. However, both Simscape and OpenSim are physics-based modeling platforms grounded in the same biomechanical principles. Any differences between them would primarily arise from their inverse kinematics algorithms, not from underlying physical laws. Introducing a second modeling platform would add algorithmic variability, making it more difficult to isolate the effect of segment length estimation—the primary focus of our study. To ensure a controlled comparison, we intentionally kept the inverse kinematics method consistent and varied only the segment scaling approach.

8. The interpretation that morphological variability contributes less than neural redundancy is overstated, given the purely kinematic analysis; causality between morphology and neural control cannot be established without electromyography.

A. We thank the reviewer for this important observation. We agree that our data are purely kinematic and do not permit direct inference about neural mechanisms. Our interpretation regarding “neural redundancy” was meant to highlight the functional implications of within-participant variability, which likely reflects the motor system’s use of redundant degrees of freedom. However, we recognize that causal attribution to neural control is not supported without accompanying neurophysiological data, such as electromyography (EMG). To address this concern, we have made the changes to avoid overstating the interpretation in Abstract on lines 49-52, Discussion on lines 308-313. We also consolidated all limitations into a single paragraph on lines 321-333 in Discussion.

9. A sentence should be added stating that while robust for reaching, the findings should be verified for other movement types (gait, whole-body tasks)

A. This limitation was added on lines 321-333.

10. The manuscript must explicitly qualify its clinical implications, as the findings are derived solely from a healthy cohort. It is recommended to add a dedicated statement in the Discussion to clarify that the direct application to clinical assessment is not yet proven. Specifically, it should be noted that in clinical populations, the relationship between segment scaling error and motor variability may be fundamentally altered due to neurological damage that changes the structure of movement.

A. We have added a limitation statement on lines 321-333.

11. Potential for altered limb morphology in clinical populations could affect segment proportions, a factor not explored in this study. Therefore, the robustness of segment scaling methods must be validated in patient groups before these findings can be confidently applied to clinical assessment.

A. We have added a limitation statement on lines 321-333.

12. The Discussion must explicitly state that the findings are specific to the studied upper-limb joints in a healthy, neurotypical population. The conclusions may not generalize to limbs, upper or lower, with structural deformities, where altered segment morphology and biomechanics could significantly amplify the error from using average segment proportions.

A. We have added a limitation statement on lines 321-333.
